# Study to Investigate the Potential of Combined Extract of Leaves and Seeds of *Moringa oleifera* in Groundwater Purification

**DOI:** 10.3390/ijerph17207468

**Published:** 2020-10-14

**Authors:** Mir Waqas Alam, Pratibha Pandey, Fahad Khan, Basma Souayeh, Mohd Farhan

**Affiliations:** 1 Department of Physics, College of Science, King Faisal University, P.O. Box 400, Hofuf, Al-Hassa 31982, Saudi Arabia; bsouayeh@kfu.edu.sa; 2 Department of Biotechnology, Noida Institute of Engineering and Technology, 19, Knowledge Park-II, Institutional Area, Greater Noida 201306 (U.P.), India; fahadkhan.bio@niet.co.in; 3 Department of Basic Science, Preparatory Year Deanship, King Faisal University, P.O. Box 400, Hofuf, Al-Hassa 31982, Saudi Arabia; mfarhan@kfu.edu.sa

**Keywords:** *Moringa oleifera*, water purification, natural coagulant, jar test apparatus

## Abstract

Several parts of the *Moringa oleifera* plant have revealed incredible potential for water quality improvement. However, the purification potential of a combined leaf and seed extract of *Moringa oleifera* plants remains unexplored. To the best of our knowledge, this research would be the first to work towards exploiting the combined potential of a leaf and seed extract of the *Moringa oleifera* plant in the process of water purification. In this study, we investigated the combined effectiveness of the leaf and seed extract in the purification of groundwater. The jar test method was used to analyze the effectiveness of *Moringa* plant extract (in combination) on different quality parameters of groundwater. Treatment with the combined plant extract (seed and leaf) resulted in significant improvement of various physicochemical (hardness, pH, turbidity, Total Dissolved Solid (TDS), and metallic impurities) and biological parameters (*E.coli* count) over individual seed and leaf extracts in groundwater samples. Experimental findings have strongly shown the enhanced purification efficacy of the hexane extract of combined plant materials in comparison to the individual extracts, thereby providing us with a potent natural coagulant that could combat the side effects of chemical coagulants.

## 1. Introduction

Globally, many populations are residing in developing countries with limited availability of pure drinking water. Approximately six million deaths of children per year have been reported due to diarrhea in developing countries [1]. Increased industrial development and population lead to the pollution of the groundwater supply, presenting several public health issues, such as waterborne diseases [1]. One of the most crucial factors of groundwater pollution is the improper drainage of untreated industrial effluents directly into water bodies and agricultural fields. These untreated industrial effluents ultimately result in an increased accumulation of heavy metals in the food chain [2]. This evidence is a source of alarming concern to these countries, necessitating the development of alternative and cost-effective methods or technologies that could solve the problem of ensuring a clean water supply.

Various conventional methods have been utilized for wastewater treatment, such as PABs (permeable adsorptive barriers), coagulation, flocculation, filtration, reverse osmosis, and ion exchange resins, which are applied before the water is distributed to consumers [3,4]. Despite the significant role of synthetic and chemical coagulants in water purification, they are associated with several neurotoxic and carcinogenic effects due to their leftover residuals in the treated water, such as aluminum [5]. Numerous reports have focused on developing eco-friendly and sustainable natural coagulants as a potential alternative to chemical coagulants in water purification [6,7,8]. Therefore, this research mainly focused on exploring the coagulation potential of the *Moringa oleifera* plant for water purification.

*Moringa oleifera* (horseradish tree) is a fast-growing and widely cultivated tree in India. Every part of the *Moringa* plant has been associated with multiple health and nutritional benefits due to the presence of numerous bioactive compounds [9,10]. Previous scientific reports have substantiated several pharmacological attributes, including anti-inflammatory, analgesic, antihypertensive, antioxidant, and anticancer properties [11,12]. In addition to the pharmacological and nutritional health benefits, the *Moringa* plant has also been recognized as a potential coagulant in water purification with no adverse side effects on public health, even at higher doses [13,14]. Furthermore, the coagulant sludge produced during the water purification process could be efficiently used as organic plant fertilizer or animal feed. Although several research findings have exhibited the role of *Moringa* seed powder as an effective natural coagulant [15,16,17], they had some limitations, such as a high sludge volume. *Moringa* leaves have also exhibited significant pharmacological properties due to the presence of a high amount of bioactive compounds, such as carotenoids, flavonoids, polyphenols, alkaloids, isothiocyanates, saponins, and tannins. Recently, we elucidated the water purification potential of *Moringa oleifera* leaf extract in comparison to alum (chemical coagulant). Treatment with leaf extract has shown a better coagulation potential than the seed extract, which motivated us to further explore the potential of the combined effect of a seed and leaf extract of *Moringa oleifera* [18,19]. Therefore, this research was designed to investigate the combined potential of *Moringa oleifera* seed and leaf extract using hexane solvent as a potent alternative to synthetic coagulants in improving groundwater quality parameters. 

## 2. Materials and Methods

### 2.1. Reagents

The organic solvent (n-hexane) used for the extraction process and other reagents were procured from HiMedia Pvt. Ltd. (Mumbai, India). All the reagents and chemicals utilized were of analytical grade. A jar test apparatus (flocculator) R A Scientific Instrument, New Delhi, India) was utilized for the evaluation of the coagulating potential of the combined leaf and seed extract of the *Moringa oleifera* plant in surface water purification.

### 2.2. Water Sample (Groundwater)

In our study, groundwater samples were collected from the Noida Institute of Engineering and Technology (NIET) Campus, situated in the Greater Noida region of Uttar Pradesh (India). The groundwater sample used in the experimental processes was collected in polyethylene plastic bottles (sterile). The treated water samples were then stored at −20 °C for future experimental use.

### 2.3. Preparation of Plant Extract

The *Moringa oleifera* plant materials (leaves and seeds) were obtained from the NIET campus. The leaves and seeds were washed and shed dried at room temperature for a minimum of 21 days. Thereafter, both plant parts were dried in a hot air oven for some time at 35 °C. Finally, the plant material was converted into a fine, coarse powder, which was further utilized for the extraction process.

### 2.4. Extraction Procedure by Soxhlet Apparatus

Plant extracts were prepared via the Soxhlet extraction method in which the organic solvent (n-hexane) was used in a ratio of 1:10 (1 part fine powder and 10 parts hexane solvent) to obtain the crude extract. Equal amounts of both plant materials (20 g each) in powder form were transferred into the extraction thimble and extracted in n-hexane (400 mL) solvent for 9 h in the Soxhlet apparatus (Borosil, Mumbai, India). This process continued until a transparent solvent was obtained [18,19]. After the extraction step, the n-hexane solvent was evaporated by drying, and the plant extracts were stored at a low temperature for further experiments.

### 2.5. Coagulation Procedure Using Flocculator Apparatus

A flocculator apparatus was used to test the coagulation efficiency of the combined *Moringa oleifera* plant extract in water purification [18,19]. In brief, different concentrations (0 mg/L, 25 mg/L, 50 mg/L, and 100 mg/L) of natural coagulants from the *Moringa oleifera* plant were added into each beaker and stirred at different speed, viz. 50 rpm for 15 min, a rapid mixing at 150 rpm for 10 min, followed by slow stirring at 50 rpm for 15 min. The obtained supernatant was settled down, filtered, and then analyzed for various physicochemical and biological parameters. This experiment was performed at room temperature.

### 2.6. Comparative Analysis of Physicochemical Parameters in Groundwater

To further confirm the purification potential of the plant extracts, the groundwater samples treated with the combined and individual extracts of *Moringa oleifera* were then analyzed for various physicochemical parameters using standard methods.

#### 2.6.1. Physicochemical Parameters

Several physical and chemical parameters, including turbidity, pH, total dissolved solids, and total hardness were evaluated in both the treated and untreated water samples as per the protocol described by Pandey et al., 2020 [19]. First, the pH was evaluated with a pH meter (electronic method) (Nihar Instruments, Mumbai, India). Further, hardness (due to the presence of anionic and cationic entities) in groundwater was estimated by the Octa Aqua Test Kit (WT023) from HiMedia Pvt. Limited, Mumbai, India [20]. Thereafter, TDS (total dissolved solids) was determined using a TDS meter by Hanna Instruments, New Delhi, India in the groundwater sample. Lastly, the effect of natural coagulants of *Moringa oleifera* on turbidity in the water sample was analyzed using the Octa Aqua Test Kit (WT023) from HiMedia Pvt. Limited, Mumbai, India.

#### 2.6.2. Metallic Impurities

Industrial effluents and sewage are some of the factors responsible for increased metallic content in groundwater [21]. To observe the effect of these natural coagulants on fluoride and iron content, the Octa Aqua Test Kit (WT023) from HiMedia Pvt. Limited, Mumbai, India, was used.

### 2.7. Biological Parameter (Escherichia coli) Analysis

The effect of the combined extract on *E. coli* count was elucidated by the protocol described earlier in [22]. Water samples treated with the *Moringa* extracts were sequentially diluted (ranging from 10^−1^, 10^−2^, and 10^−3^) with 0.1% sterile peptone water (HiMedia Laboratories, Mumbai, India). Then, the water samples were filtered with the help of 0.45 μm membrane filters and poured on Petri plates (Borosil, Mumbai, India) containing MLGA (membrane lactose glucuronide agar) (HiMedia Laboratories, Mumbai, India). These Petri plates were incubated at 37 °C for 1 day, and the resultant *E. coli* count (green colonies) was quantified by a count/100 mL.

### 2.8. Statistical Analysis

Experiments were repeated three times (in triplicates), and the obtained data were shown as mean ± SEM (Standard Error Mean). Statistical analysis was done using ANOVA (one-way analysis of variance), and differences were considered statistically significant at *p*-values less than 0.05.

## 3. Results

### 3.1. Effect of the Moringa oleifera Plant Extract on pH

**Figure 1:**
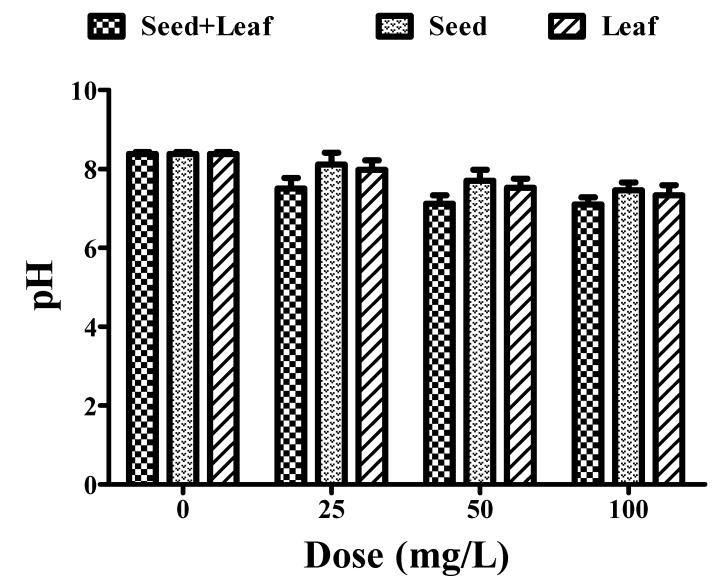
Comparative experimental analysis of combined (seed and leaf) and individual extracts of the *Moringa oleifera* plant on pH parameters of groundwater at different doses (25–100 mg/L). All the experiments were done three times in triplicates and presented as mean ± SEM (Standard Error Mean).

The permissible limit of pH lies in the range of 7–8 for drinking water [23]. Treatment of water samples with the individual leaf and seed extracts of *Moringa oleifera* led to a remarkable change in pH in a dose-dependent manner. However, the combined extracts showed better efficacy in the pH reduction (8.38 to 7.10) than the individual extracts of seeds (8.38 to 7.46) and leaves (8.38 to 7.33) (Figure 1, Table 1). Therefore, these experimental findings clearly indicate that the combined extract has a greater potential for pH reduction in comparison to the individual seed and leaf extracts of *Moringa oleifera*, and should thus be further utilized for water purification.

### 3.2. Effect of the Moringa oleifera Plant Extract on TDS

**Figure 2:**
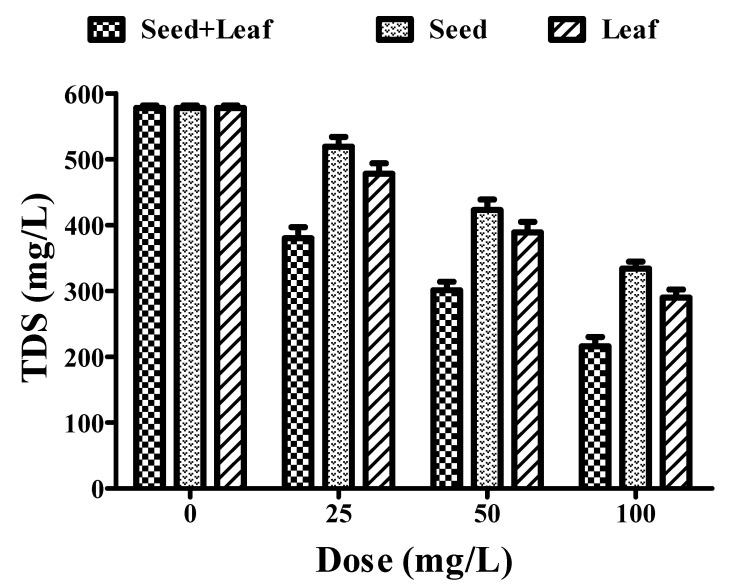
Comparative experimental analysis of combined (seed and leaf) and individual extracts of the *Moringa oleifera* plant on TDS parameters of groundwater at different doses (25–100 mg/L). All the experiments were done three times in triplicates and presented as mean ± SEM.

Furthermore, the TDS removal efficiency of both the combined and individual extracts of *Moringa oleifera* plant was analyzed. As shown in Figure 2, individual treatment with leaf (578 to 290 mg/L) and seed (578 to 334 mg/L) extracts presented moderate reductions in the TDS of the groundwater samples. However, the combined treatment (578 to 216 mg/L) resulted in a significant reduction in TDS as compared to individual treatments at similar doses (25–100 mg/L) (Figure 2, Table 1). These findings strongly validate the strong coagulation potential of the combined extract compared to the individual extracts of *Moringa oleifera* seeds and leaves.

### 3.3. Effect of Moringa oleifera Plant Extract on Total Hardness

**Figure 3:**
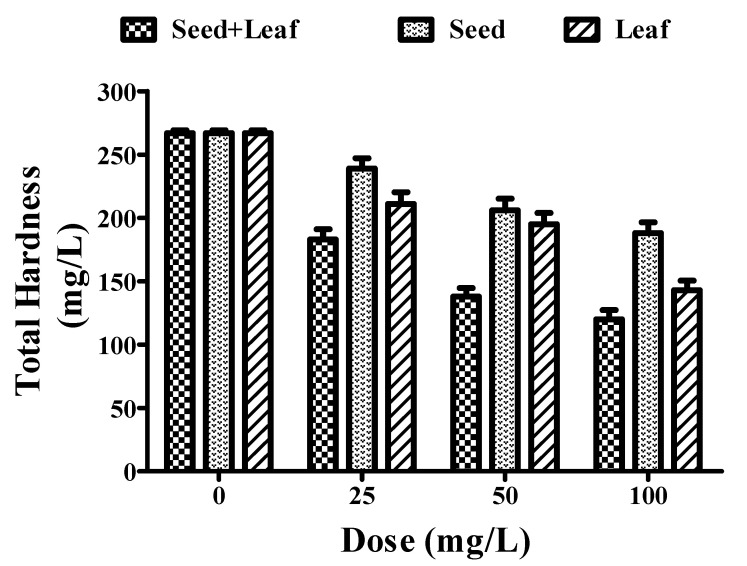
Comparative experimental analysis of combined (seed and leaf) and individual extracts of the *Moringa oleifera* plant on the hardness of groundwater at different doses (25–100 mg/L). All the experiments were done three times in triplicates and presented as mean ± SEM.

The combined extract of the *Moringa oleifera* plant led to a more prominent decrease in the total hardness of the groundwater sample (Figure 3, Table 1) in comparison with the individual seed and leaf extracts. It can be seen in Figure 3 that there was a significant change in the total hardness of the water sample treated with the combined extract (267 to 120 mg/L) at a concentration of 25–100 mg/L. Additionally, individual treatments with the leaf extract (267 to 143 mg/L) showed a better potential than the seed extract (267 to 188 mg/L) at a dose of 0, 25, 50, and 100 mg/L.

### 3.4. Effect of Moringa oleifera Plant Extract on Turbidity

**Figure 4:**
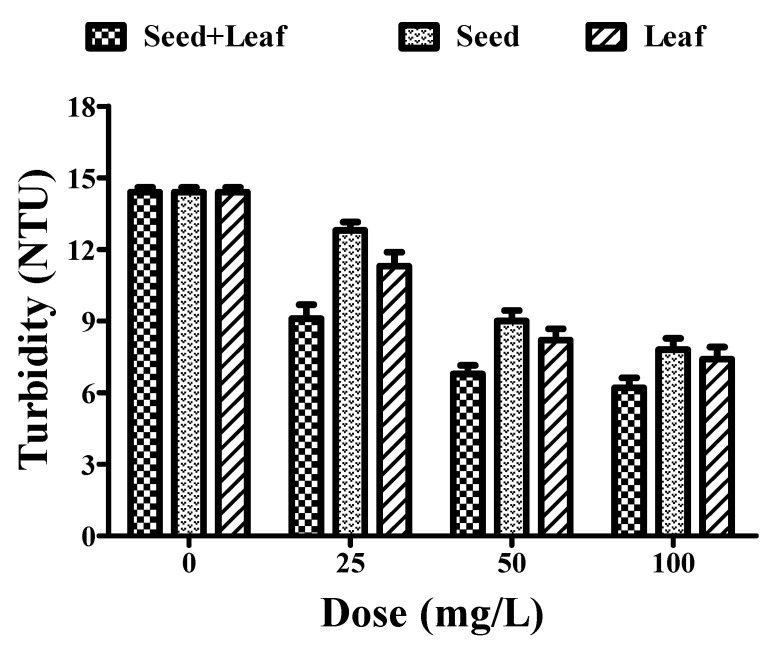
Comparative experimental analysis of the combined (seed and leaf) and individual extracts of the *Moringa oleifera* plant on turbidity parameters of groundwater at different doses (25–100 mg/L). All the experiments were done three times in triplicates and presented as mean ± SEM; NTU: Nephelometric Turbidity Unit.

The turbidity of groundwater samples treated with the individual extracts of seeds (14.4 to 7.8 NTU) and leaves (14.4 to 7.4 NTU) of *Moringa oleifera* at a dose range of 25–100 mg/L was reduced in a dose-dependent manner (Figure 4, Table 1). However, treatment with the combined extract (14.4 to 6.20 NTU) led to a more prominent reduction in the turbidity at a similar dose range of 25–100 mg/L for the individual seed and leaf extracts of the *Moringa oleifera* plant, thereby revealing a better turbidity removal efficiency of the combined extract.

### 3.5. Effect of Moringa oleifera Plant Extract on Metallic Impurity

**Figure 5:**
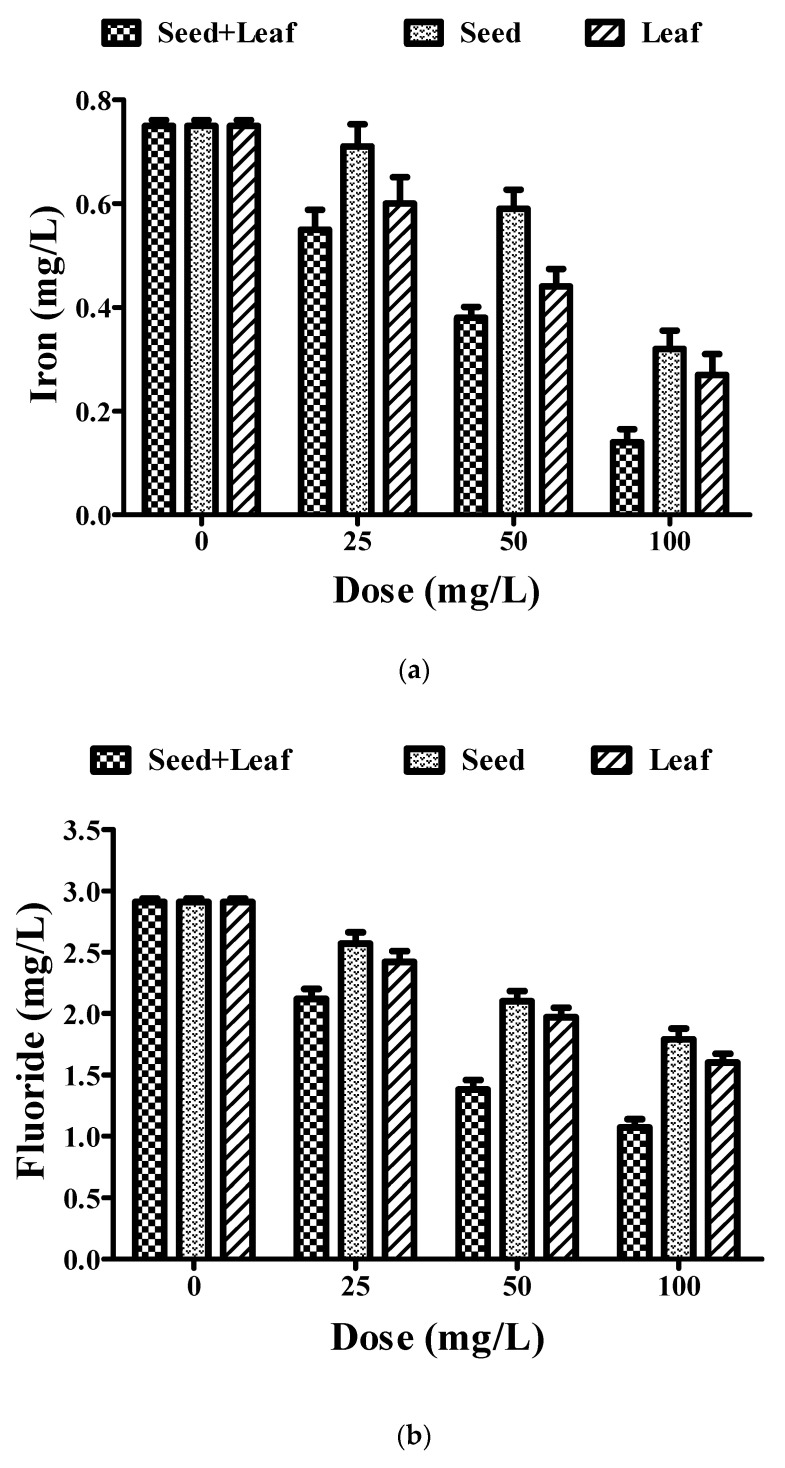
Comparative experimental analysis of combined (seed and leaf) and individual extracts of *Moringa oleifera* plant on metallic impurity parameters such as (**a**) iron and (**b**) fluoride in groundwater at different doses (25–100 mg/L). Experiments were done three times in triplicates and presented as mean ± SEM.

The water sampling site selected for this study has been mainly associated with increased fluoride and iron impurities. Therefore, we mainly focused our study to analyze the effect of *Moringa oleifera* plant extracts on these two metallic impurities in groundwater. Both the individual and combined extracts of *Moringa oleifera* led to a prominent reduction in iron and fluoride contents at doses of 0, 25, 50, and 100 mg/L. However, combined seed and leaf extract was more effective in reducing the levels of iron and fluoride in the groundwater (Figure 5, Table 1).

### 3.6. Effect of the Moringa oleifera Plant Extract on Biological Contaminant (E. coli)

**Figure 6:**
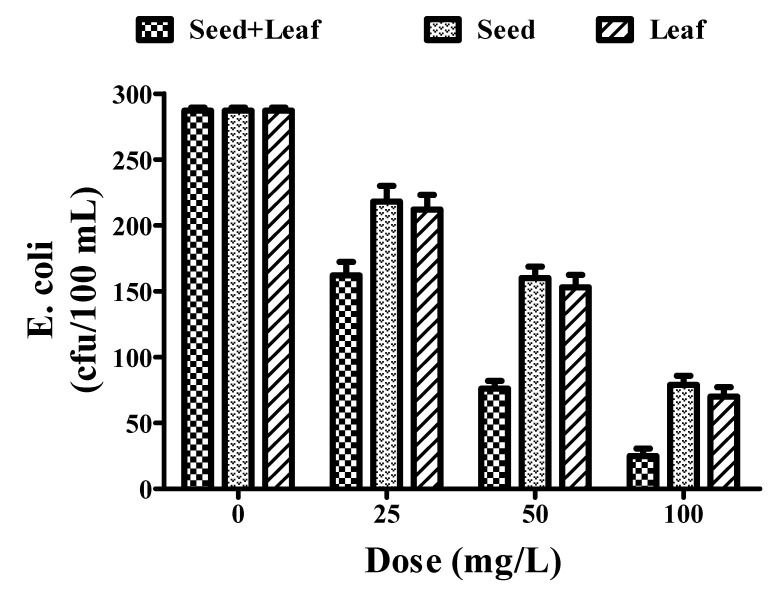
Comparative experimental analysis of combined and individual extracts of the *Moringa oleifera* plant on *E. coli* count in groundwater at different doses (25–100 mg/L). All experiments were done three times in triplicates and presented as mean ± SEM.

Numerous studies have revealed the antimicrobial potential of several parts of the *Moringa oleifera* plant. Therefore, we analyzed the antimicrobial efficacy of the plant extracts on the *E. coli* count. In the present study, the effects of both the combined and individual seed and leaf extracts of the *Moringa oleifera* plant on a biological pollutant (i.e., *E. coli*) in the groundwater sample were evaluated. The combined treatment (287 to 25 cfu/100 mL) (cfu: colony forming unit) of the groundwater sample led to a significant reduction in the *E. coli* count compared to the individual extracts of seeds (287 to 79 cfu/100 mL) and leaves (287 to 70 cfu/100 mL) of the *Moringa oleifera* plant (Figure 6, Table 1).

## 4. Discussion

Natural coagulants have gained major attention in water treatment processes thanks to their several benefits, including their eco-friendly, sustainable nature and low cost [24,25]. Natural coagulants have shown a strong potential over conventional, synthetic, and inorganic coagulants, and have become the primary focus of water operators in sustainable water treatment facilities [26]. Additionally, the positive attributes of these coagulants (green chemicals) are their limited side effects on the environment, and their isolation from natural and renewable resources provides additional benefits in reducing the costs associated with sludge disposal [27,28]. Traditional methods of coagulation or flocculation make use of synthetic or chemical coagulants such as aluminum sulfate (alum) and iron salts, but they pose serious health hazards to humans, such as neurodegenerative and gastrointestinal disorders [29].

In the past several years, the utilization of *Moringa oleifera* seeds in water purification was extensively studied due to the presence of cationic proteins (dimeric), responsible for their anticoagulation potential [30,31,32,33,34,35]. Although both the seed and leaf extracts have shown significant coagulation potential in water treatment processes, no study has reported the coagulation potential of the combined seed and leaf extract of the *Moringa oleifera* plant. Therefore, in this study, we identified the coagulation potential of the combined seed and leaf extract of the *Moringa oleifera* plant in the water purification process. To analyze the coagulating efficiency of the combined extract of *Moringa oleifera*, we estimated the levels of several quality parameters in treated water samples, including physicochemical and biological parameters. Recent experimental findings have reported that the use of alum in water treatment operations resulted in an altered pH, whereas the pH of water treated with *Moringa oleifera* extracts remains unaffected [36,37]. Our study also corroborated these findings and showed that treatment with the combined extract of *Moringa oleifera* resulted in a reduction of the pH to its normal value, as per the WHO standards (6.0–8.5). Numerous reports have explained that a higher level of TDS (total dissolved solids) poses severe side effects to humans, mainly decreased palatability and gastric abnormalities [38,39]. In accordance with previous research, the present study also proved the coagulating efficacy of the seed and leaf extracts (either individually or in the combined form) in reducing the dissolved solids. Another parameter responsible for poor water quality is turbidity, which increases either due to sediment resuspension or improper filtration of fine particles and colloidal impurities. These impurities lead to anticoagulation stability, which can only be disturbed by freezing, heating, application of a magnetic field, or application of electrolytes [40,41]. Several studies identified the presence of polyelectrolytes in the seeds of *Moringa oleifera* that might be helpful in the removal of turbidity in groundwater [42,43]. In this study, we also showed that treatment with the plant extracts exhibited significant turbidity removal efficiency during the water purification process, in accordance with the reported literature [44]. The *Moringa oleifera* seed extract also has an important ability to remove ions (softening agent) responsible for hardness in water [44]. Acceptable limits of hardness in groundwater for public use should be in the range of 300–500 mg/L. Treatment with the combined extract resulted in a significant reduction in the hardness of the water sample, which was in accordance with previous literature [45,46].

We have also focused our research on exploiting the potential of the plant extracts in the removal of metallic impurities found in groundwater, such as iron and fluoride. These impurities have been crucially associated with health hazards to humans, such as fluorosis [47]. The combined treatment resulted in a significant reduction in the levels of iron and fluoride (as per WHO standards) in comparison to the individual seed and leaf extracts of the *Moringa oleifera* plant. Another major concern associated with reduced water quality in developing countries is microbial contamination, which results in several waterborne diseases, such as diarrhea [48,49]. In this study, the seed and leaf extract of *Moringa oleifera* showed a remarkable reduction in the *E. coli* content of the water, thereby validating it as an effective natural coagulant in improving the water quality index. Previous reports have explained the possible mechanism behind the efficacy of the *Moringa* seed extract in lowering the *E. coli* count as being a result of its destabilization of colloidal particles. The decrease in the *E. coli* count in our research findings was in accordance with these previous reports. Hence, it could be hypothesized that the possible mechanism behind the antimicrobial potential of these extracts could be due to precipitation or cell membrane disruption [50,51]. Altogether, the combined extract (seed and leaf) showed a better coagulation potential than the individual seed and leaf extracts of *Moringa oleifera*, improving various water quality parameters.

## 5. Conclusions

Natural coagulants are currently in demand due to their numerous health benefits and minimal toxicity. Thus, we elucidated the coagulating potential of the *Moringa* plant in water purification. The novel rationale behind this research was to find the water purification potential of leaf and seed extracts in combination and their comparison with the individual extracts. Our results clearly indicate that combined treatment can significantly enhance the coagulation potential of these individual extracts. Thus, this research strongly concludes that the combined seed and leaf extract obtained in a hexane solvent might be considered as a potent natural coagulant with minimal or no side effects. Further studies are needed to exploit the potential of the combined seed and leaf extract of the *Moringa oleifera* plant in developing a cost-effective technology for water purification.

## Figures and Tables

**Table 1:** Comparison of the effects of different plant parts of *Moringa oleifera* (seeds and leaves) on groundwater.

S. No.	Water Quality Parameters	Untreated Water	Treated Water								
			25 mg/L			50 mg/L			100 mg/L		
			S + L ^a^	S ^b^	L ^c^	S + L	S	L	S + L	S	L
**1**	pH	8.38 ± 0.03	7.50 ± 0.23	8.11 ± 0.20	7.98 ± 0.18	7.12 ± 0.28	7.70 ± 0.18	7.52 ± 0.193	7.10 ± 0.22	7.46 ± 0.21	7.33 ± 0.310
**2**	TDS * (mg/L)	578.00 ± 3.11	380.00 ± 10.99	519.00 ± 12.99	478.00 ± 11.98	301.00 ± 13.98	423.00 ± 10.98	389.00 ± 11.12	216.00 ± 11.34	334.00 ± 14.60	290.00 ± 14.29
**3**	Hardness (mg/L)	267.00 ± 3.11	183.00 ± 6.99	239.00 ± 9.23	211.00 ± 9.87	138.00 ± 9.10	206.00 ± 7.98	195.00 ± 7.72	120.00 ± 7.21	188.00 ± 7.60	143.00 ± 9.10
**4**	Turbidity (NTU ^#^)	14.40 ± 0.12	9.10 ± 0.68	12.80 ± 0.52	11.30 ± 0.46	6.78 ± 0.43	9.00 ± 0.48	8.20 ± 0.36	6.20 ± 0.39	7.80 ± 0.36	7.40 ± 0.40
**5**	Fluoride (mg/L)	2.91 ± 0.02	2.12 ± 0.09	2.57 ± 0.08	2.42 ± 0.07	1.38 ± 0.10	2.10 ± 0.06	1.97 ± 0.06	1.07 ± 0.07	1.79 ± 0.08	1.60 ± 0.09
**6**	Iron (mg/L)	0.75 ± 0.02	0.55 ± 0.09	0.71 ± 0.08	0.60 ± 0.07	0.38 ± 0.10	0.59 ± 0.06	0.44 ± 0.06	0.14 ± 0.07	0.32 ± 0.08	0.27 ± 0.09
**7**	*E. coli* (cfu ^$^/100 mL)	287.00 ± 0.98	162.00 ± 10.10	218.00 ± 7.89	212.00 ± 9.12	76.00 ± 8.78	160.00 ± 8.98	153.00 ± 9.11	25.00 ± 6.99	79.00 ± 8.19	70.00 ± 6.12

^a^ S + L: Seed + Leaf; ^b^ S: Seed; ^c^ L: Leaf; * TDS: Total dissolved solids; ^#^ NTU: Nephelometric turbidity unit; ^$^cfu: colony forming unit; Results presented as mean ± SEM.
